# Acute Myocardial Infarction in the Setting of Pulmonary Hypertension due to a Patent Foramen Ovale and Paradoxical Embolism

**DOI:** 10.1155/2024/6725308

**Published:** 2024-07-18

**Authors:** Madeline Franke, Zeenat Safdar

**Affiliations:** ^1^ School of Engineering Medicine Texas A&M Health Science Center, Houston, Texas, USA; ^2^ Houston Methodist Lung Center Houston Methodist Hospital Weill Cornell College of Medicine, Houston, Texas, USA

**Keywords:** myocardial infarction, paradoxical embolism, patent foramen ovale, pulmonary hypertension, shunt, thrombus, troponin

## Abstract

A 67-year-old woman with pulmonary hypertension (PH) presented with a 1-day history of worsening shortness of breath and pleuritic chest pain and was found to have a troponin T level of 3755 ng/L (ref. range 0–19 ng/L). An initial diagnostic workup in the emergency department (ED) led to an urgent left heart catheterization which revealed a 90% occlusive right coronary artery blood clot, even though a recent heart catheterization less than a month prior was completely unremarkable. Further workup led to the discovery of a patent foramen ovale (PFO) and an aneurysmal interatrial septum, suggesting the presence of a paradoxical embolism. While typically asymptomatic, a PFO is an important clinical entity that can lead to irreversible cardiac damage. Suspicion should be high for this finding in the case of an acute myocardial infarction (MI) with no clear cause, especially in a patient with elevated right heart pressures.

## 1. Introduction

Acute myocardial infarction (MI) is a life-threatening condition with a global prevalence of almost 3 million [1]. Resulting from an abrupt decrease in blood flow to the myocardium, an MI can lead to irreversible cell death and long-term damage to the heart [2]. Most cases of acute MI are due to long-standing coronary artery disease (CAD), associated with factors such as smoking, hypertension, diabetes mellitus, and obesity [2]. Typically, patients present with left-sided chest discomfort or pressure that can radiate to the left neck, jaw, or arm [2]. However, atypical presentation with nonspecific symptoms, such as musculoskeletal pain, has been described [3–5]. In such instances, it is important to distinguish between common musculoskeletal pain and an acute MI, and prompt recognition allows for the early initiation of life-saving treatment [3, 4]. Atypical MIs can present in patients with few risk factors or with no prior history of CAD, and in such cases, paradoxical coronary embolisms and other acute causes for the blockage of coronary blood flow should be considered [6].

An uncommon, yet important, cause of an acute MI is paradoxical coronary embolism through a patent foramen ovale (PFO) [4, 6]. A PFO is a common congenital heart defect caused by the failed closure of a small embryonic opening between the left and right atria, allowing unidirectional blood flow from the right to the left side [7, 8]. Persisting in up to 35% of adults worldwide, this abnormality can be clinically silent in most patients [9, 10]. However, in the presence of elevated right heart pressures, such as in pulmonary hypertension (PH), shunting of blood flow from the right to the left side of the heart can be heightened from the patient's physiological baseline [11, 12]. This passage of blood bypassing the pulmonary system can lead to paradoxical emboli from the venous to the arterial system [6, 7, 13]. Although such cases are not common, determining the cause of the acute event is critical for guiding prompt intervention and management.

Our case highlights the importance of recognizing a PFO as a potential cause of MIs in an atypical patient presentation. Furthermore, our case features markedly elevated troponin levels and demonstrates how further investigation is warranted if clinical suspicion for a PFO and paradoxical embolism is high.

## 2. Case Presentation

A 67-year-old African American woman with a history of PH, heart failure with preserved ejection fraction, chronic kidney disease, essential hypertension, gastroesophageal reflux disease, prior gastric bypass surgery complicated by a gastrointestinal bleed secondary to gastrojejunal anastomotic ulceration, and previous deep vein thrombosis (not on anticoagulation) presented to the emergency department (ED) with shortness of breath. The patient reported that she was in her normal state of health until 1 day before admission when she started experiencing shortness of breath at rest with no precipitating event. Associated symptoms included acute, 7/10 nonradiating, centralized chest pain, which worsened with deep inhalation. She denied any constitutional symptoms, such as fever, chills, nausea, vomiting, palpitations, dizziness, or malaise.

Of note, this patient was being worked up for PH by our team and carried a presumed diagnosis of Group 4 chronic thromboembolic PH (CTEPH). Before presenting to our clinic, she was initially diagnosed with PH by an outside facility after experiencing worsening dyspnea on exertion and wheezing following a November 2022 COVID-19 infection. She had no prior history of breathing problems, history of PH, or similar symptoms prior to this COVID-19 diagnosis. At this time, the patient was treated symptomatically and was not started on any long-term therapy for her PH. She was then referred to our PH center for further workup and management. Our initial clinical workup included a computerized tomography scan of the chest and a V/Q perfusion scan which suggested an underlying chronic pulmonary embolism and CTEPH as possible causes of her symptoms. A transthoracic echocardiogram (TTE) showed normal systolic function with an ejection fraction of 60%–65% and right ventricular systolic pressure of 64 mmHg. Due to her concern for CTEPH, she underwent a pulmonary angiogram with right and left heart catheterization in July 2023. A coronary artery angiogram was also performed during the left heart catheterization to assess for vessel blockages. These procedures demonstrated severely elevated right filling pressures but revealed patent coronary arteries with no occlusions. Specifically, this catheterization revealed moderate PH and suggested CTEPH as an etiology for her PH. Hemodynamics are presented in Table 1 with corresponding normal values for each data point [14]. Given the patient's history of gastric bypass surgery complicated by a gastrointestinal bleed, the gastroenterology team was consulted to obtain clearance prior to starting her on long-term anticoagulation. Following the pulmonary angiogram, she was started on riociguat, 0.5 mg three times a day, and the dose gradually increased from 0.5 mg every 8 h to 2.0 mg every 8 h. This last dose change happened on the same day that she started experiencing shortness of breath and chest pain.

Upon arrival at the ED, she was hemodynamically stable with a heart rate of 76 beats/min, respiratory rate of 18 breaths/min, blood pressure of 116/71 mmHg, and oxygen saturation of 91% on room air. Physical examination revealed tachypnea and increased respiratory effort, with noted intracostal contractions and some basilar crackles. Jugular venous distention and bilateral lower extremity edema were present. A systolic murmur was appreciated at the upper sternal border. Initial labs were notable for a troponin T level of 3755 ng/L (normal < 19 ng/L) and an N-terminal prohormone of brain natriuretic peptide (NT-proBNP) of 2053 pg/mL (normal < 125 pg/mL). Troponin T and NT-proBNP levels over time are shown in Table 2.

**Figure 1:**
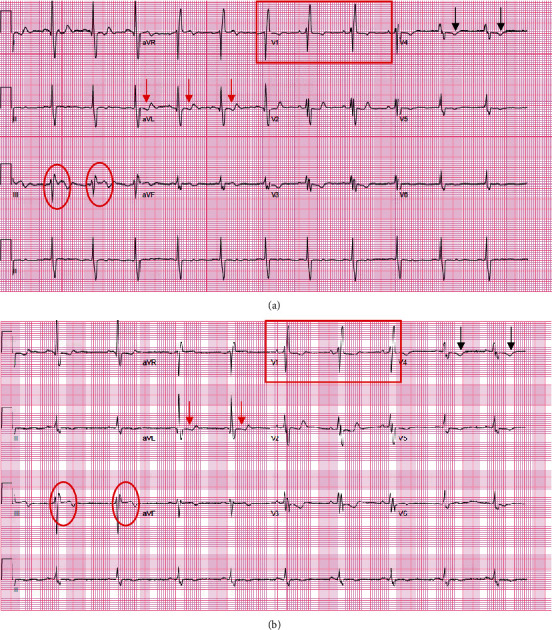
(a) Initial EKG done in the emergency department showed normal sinus rhythm; an old right bundle branch block (outlined with red box); mild ST depressions (< 1 mm) in leads I, aVL (denoted with red arrows in aVL); T-inversions in II, V4–5 (denoted with black arrows in V4); and ST elevation in III, aVF (< 1 mm) (denoted with red circles in lead III). (b) EKG done 25 min later shows similar findings, but sinus bradycardia.

A 12-lead electrocardiogram (EKG) performed in the ED did not reveal any clear acute process and was similar to prior EKGs from previous admissions (Figure 1). A chest X-ray was indicative of an enlarged pulmonary artery and cardiac silhouette, along with minimal atelectatic changes in the bilateral lung bases. A computerized tomography angiogram of the chest was negative for an acute pulmonary embolism and unchanged mosaicism in both lungs. A bilateral lower extremity Doppler ultrasound showed no evidence of deep venous thrombosis in the visualized lower extremity veins.

**Figure 2:**
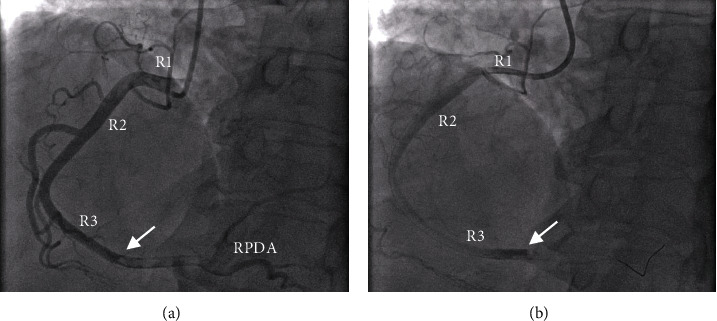
Left heart catheterization images depict a blockage in the distal right coronary artery. Each image shows the blockage from a left anterior oblique (LAO) view with (a) cranial angulation and (b) caudal angulation. For both (a) and (b), the white arrow points to the vessel blockage. R1 = proximal right coronary artery; R2 = middle right coronary artery; R3 = distal right coronary artery; RPDA = right posterior descending artery.

Given the remarkably elevated troponin T levels and symptomatic chest pain, she underwent repeat left heart catheterization (Figure 2). A large, 1.5-in. thrombus occluding 90% of the dominant, distal right coronary artery was noted, and mechanical thrombectomy was performed. Blood flow to the right coronary artery was restored, and repeat EKGs did not reveal any new changes. Following the thrombectomy, her chest pain and shortness of breath resolved to her baseline, and she remained hemodynamically stable.

**Figure 3:**
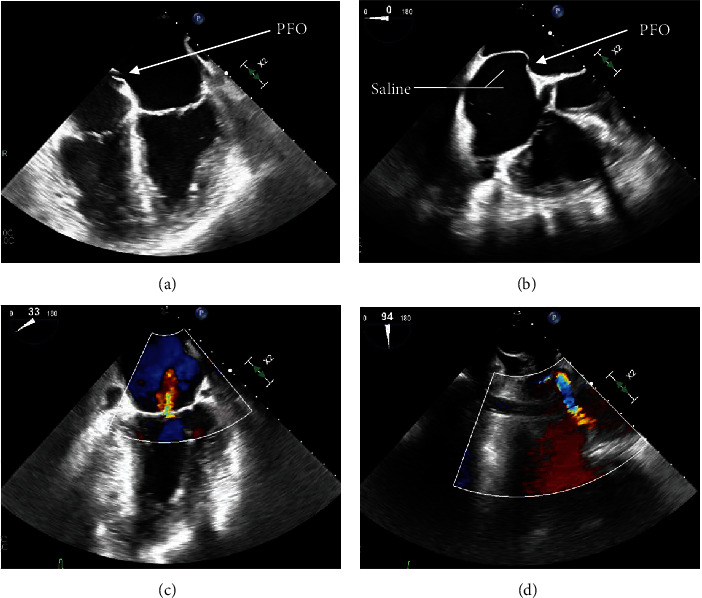
Transesophageal echocardiography revealing the patent foramen ovale (PFO). (a, b) The PFO (white arrow) in different views. (b) The leakage of saline contrast across the atria. (c, d) The shunting/mixing of blood through the PFO.

Further diagnostic workup to determine the etiology of the occlusive right coronary artery clot was undertaken, considering the left and right heart catheterization done a few days prior to admission did not show any evidence of thrombi or significant CAD. The transesophageal echocardiogram (TEE) with color flow Doppler and intravenous saline contrast revealed a right-to-left shunt through a PFO, along with an aneurysmal interatrial septum (Figure 3). No atrial or ventricular thrombus or mass was visualized.

**Figure 4:**
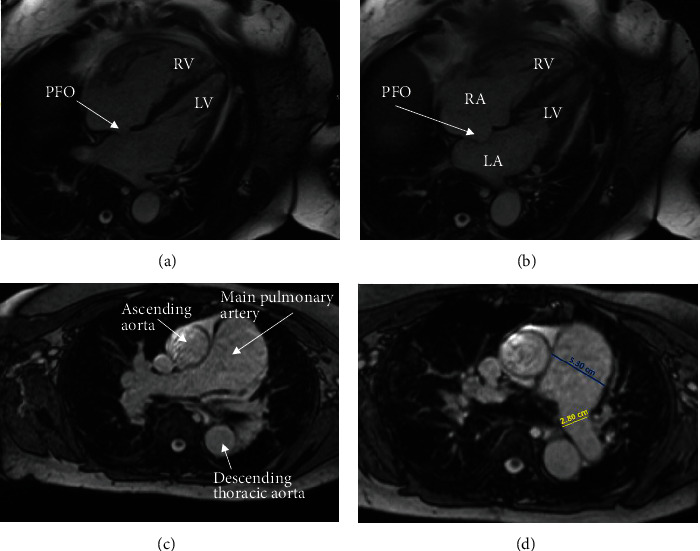
Cardiac MRI revealing the patent foramen ovale (PFO). (a, b) The PFO (white arrow) and interatrial aneurysm. (c, d) The dilated main pulmonary artery. In (d), the blue line denotes the main pulmonary artery (5.30 cm), and the yellow line denotes the left pulmonary artery (2.80 cm). LA, left atrium; LV, left ventricle; RA, right atrium; RV, right ventricle.

A cardiac magnetic resonance imaging (MRI) scan confirmed the presence of an atrial septal aneurysm, which is defined as a rare cardiac deformity where the septum bulges into one side of the atria, along with a PFO (Figure 4) [15]. The hypercoagulability workup was negative for a Factor V Leiden mutation, prothrombin gene mutation, von Willebrand disease, protein C and S deficiencies, and cardiolipin and beta-2 glycoprotein antibodies. Additionally, a full connective tissue disease workup was done, including lupus anticoagulant, antinuclear antibodies, cyclic citrulline peptide antibodies, double-stranded DNA antibodies, Jo-1 antibodies, rheumatoid factor, ribonucleic antibodies, scleroderma SCL-70 antibodies, Sjogren's SS-A/SS-B antibodies, Smith antibodies, and inflammatory markers (C-reactive protein, erythrocyte sedimentation rate, and ferritin). None of these studies revealed an underlying connective tissue or autoimmune disease.

Following the coronary thrombectomy, our patient was treated with double anticoagulation consisting of a heparin drip and clopidogrel. Plans for the transition from heparin drip to apixaban were made upon discharge for long-term anticoagulation. The patient would require life-long anticoagulation as well as treatment to control her CTEPH with riociguat and the addition of macitentan. Unfortunately, PFO closure was not recommended for our patient due to her complex medical history and risk of worsening right heart failure. The treatment was therefore focused on careful medical surveillance and controlling her CTEPH/PH, anticoagulation, and other medical problems.

The patient experienced resolution of her presenting symptoms after her thrombectomy and medical treatments. Outpatient follow-up was planned with cardiology, pulmonology, hematology, and her primary care physician.

## 3. Discussion

The final diagnosis for our patient was a non-ST-elevation MI due to acute occlusion of the distal right coronary artery, likely due to a paradoxical embolism that passed through an incidental PFO. The exact starting location of the occluding embolus could not be determined with certainty, but the presence of a PFO suggests that the emboli bypassed the pulmonary circulation. To cause an MI, the thrombus would have had to pass from its location of origin through the PFO, ending up entering the arterial circulation from the venous circulation. Once in the arterial circulation on the left side of the heart, the thrombus would have to leave through the aorta and enter the dominant coronary circulation, which in our case was the right coronary artery. In our patient, the clot was found occluding the distal right coronary artery, where the thrombus could not travel any further.

A paradoxical embolism traveling through a PFO is an uncommon cause of MI [16, 17]. A recent retrospective and prospective combination study found that less than 1% of acute MIs are due to emboli traveling through a PFO [17]. Although infrequent, it is important to identify patients at risk to provide them with the appropriate treatment and long-term care. Since these patients do not usually have any history of CAD, the diagnosis can be missed, leading to a delay in intervention. There have been multiple reports of patients dying from a fatal coronary artery occlusion due to a paradoxical embolism traveling through interatrial communication [18, 19]. Additionally, there have been cases of individuals with a PFO who have simultaneous MI from paradoxical emboli and a pulmonary embolism [20, 21]. In such patients, it is of even greater importance to discriminate the causes of their symptoms so that the PFO and paradoxical embolism are not missed in the setting of a massive pulmonary embolism. Patients with pulmonary embolisms are more prone to paradoxical emboli through a PFO [20]. The pulmonary embolism can cause increased right heart pressures, which can allow the opening of the closed PFO with the passage of the emboli. Finally, it is important to note that paradoxical emboli traveling through a PFO affect more than just the coronary vasculature. Paradoxical emboli are a significant cause of some types of strokes, especially in young, healthy patients. A suggested 40% of strokes are cryptogenic in nature and are thought to be due to paradoxical emboli traveling through defects in the cardiac or pulmonary systems [22].

Our patient did not have an acute pulmonary embolism leading to her current presentation but still had elevated right heart pressures due to her PH which contributed to the opening of the PFO and the passage of a paradoxical embolism. A PFO is generally a one-way, unidirectional shunt from the right to the left side of the heart and is normally asymptomatic as most individuals have higher pressures on the left side of the heart [7]. However, patients with PH have elevated right heart pressures, leading to the shunting of blood through the PFO. Since PFOs have been identified in up to a quarter of patients with PH, this is an important consideration for clinicians when encountering a PH patient [23].

There are five groups of PH: pulmonary arterial hypertension (Group 1), left heart disease (Group 2), lung disease and/or chronic hypoxia (Group 3), CTEPH (Group 4), and miscellaneous/multifactorial (Group 5) [24]. The different groups of PH guide overall patient management, so it is critical to try and determine the group that a patient best fits into. Our patient likely had Group 4 CTEPH, which is a type of PH that occurs when there is an accumulation and nonresolution of many thromboemboli in the pulmonary vasculature [25]. Her computerized tomography scan of the chest revealed patchy mosaic attenuation throughout the lungs, and her perfusion scan showed multiple small defects in both lungs and worsening defects in the left lung, suggesting an underlying chronic pulmonary embolism. Additionally, her workup for causes of primary Group 1 PH was unremarkable, and she did not have any evidence of left heart disease. Interestingly, there was a discrepancy between the left ventricular end-diastolic pressure (LVEDP) and pulmonary capillary wedge pressure (PCWP) on her baseline right heart catheterization. In many cases, the PCWP is a good estimation of the LVEDP [26]. However, in our patient, the LVEDP was close to normal, while there was an isolated elevation of the PCWP. This is because the PCWP measurement is different from LVEDP in that it is an indirect measurement that incorporates the compliance of the left heart and pulmonary vasculature [27]. Determining the PCWP requires proper catheter position in the mid or lower lung zone, and over- or underwedging of the balloon can result in an erroneous reading. In contrast, the LVEDP is a direct measure that only considers the compliance of the left ventricle, and it is considered to supersede the PCWP reading [27].

Our patient presented with dyspnea and acute chest pain, which could have been due to many conditions, including worsening PH, CAD, or a repeat pulmonary embolism. Her initial clinical presentation, along with the focused physical exam findings of intracostal contractions, basilar crackles, jugular venous dilation, and bilateral lower extremity edema, put her at a higher risk for more serious disease, prompting quick decision-making and acute management [28]. Additionally, due to her prior history of DVT, pleuritic chest pain, and vague symptoms, tests were done to rule out an acute PE. Distinguishing between the presentation of acute PH and acute coronary syndrome is important in order to provide the appropriate treatment to the patient. Likewise, careful decision-making is required for patient-centered treatment as well [29]. PFO closure, while desired, carries an increased risk of heart failure progression in certain patients [30]. Since our patient had a right heart strain due to her CTEPH, it was not recommended for her to undergo corrective surgery [31]. She was therefore managed medically and followed up closely by her cardiology, pulmonary, hematology, and primary teams.

The imaging procedure of choice for PFO diagnosis is a TEE [32]. Superior to a TTE, a TEE can detect intricate cardiac morphology and be used with a saline contrast medium to visualize any contrast leakage across the atria during Valsalva. In our case, a TEE was used to ultimately diagnose our patient with a PFO, followed by a cardiac MRI to investigate the results further. It is not clear how much blood passed through the PFO at the patient's resting state or the actual size of the PFO, but we know there must have been some spontaneous communication to allow for the passage of a thrombus through the right-to-left shunt. Because of her PH, our patient had received many TTEs routinely in the past, none of which revealed the presence of a PFO. This highlights the importance of using the proper diagnostic techniques to make the correct diagnosis when a PFO is suspected.

Finally, our case describes notably high troponin T levels that were sustained over time. Our patient presented with a troponin T level of 3755 ng/L (normal < 19 ng/L) which initially alarmed clinicians and prompted further investigation. The troponin T was rechecked shortly after to make sure there was no laboratory error and was periodically measured throughout the duration of the patient's hospitalization. Elevated troponin levels indicate cardiac damage, and testing is an important part of the diagnostic workup for acute coronary syndromes. Although troponin levels can be elevated in both cardiac and noncardiac conditions, very high troponin levels are suggestive of an acute coronary process [33]. While the exact troponin cut-off level for an acute coronary process has not been defined, it has been suggested that 189 ng/L is the upper limit of normal for patients who are not diagnosed with an MI or related condition [34]. Consequently, it is important to have high clinical suspicion for an MI with such elevated troponin T levels, even if the patient presents atypically.

## 4. Conclusion

This case illustrates an uncommon complication of a usually asymptomatic clinical entity. Our patient with elevated right heart pressures was determined to have a PFO after high clinical suspicion led to further investigation. Our case highlights the importance of recognizing paradoxical embolism as a potential cause of acute MIs, especially in a patient with no previous history of coronary disease. It is critical to identify these patients quickly in order to provide them with appropriate treatment and care.

## Figures and Tables

**Table 1:** Baseline hemodynamic measurements obtained before admission to the emergency department.

**Swan–Ganz parameters**	**Baseline (8 months before admission)**	**Follow-up (27 days before admission)**	**Reference range**
RAP (mmHg)	6	13	2–6
mPA (mmHg)	40	40	9–18
PCWP (mmHg)	29	18	4–12
LVEDP (mmHg)	7	14	4–12
CO (L/min)	3.63	5.1	4.0–8.0
PVR (WU)	9.1	5.1	< 3.125

Abbreviations: CO, cardiac output; LVEDP, left ventricular end-diastolic pressure; mPA, mean pulmonary artery pressure; PCWP, pulmonary capillary wedge pressure; PVR, pulmonary vascular resistance; RAP, mean right atrial pressure; WU, wood units.

**Table 2:** Troponin T and N-terminal prohormone of brain natriuretic peptide (NT-proBNP) levels over time.

Time since arrival (hours)	1	2	6	28	48	68	92	100
Troponin T level (ng/L, ref. range 0–19)	3755	3850	3476	2939	—	2627	—	1848
NT-proBNP level (pg/mL, ref. range 0–125)	2053	—	—	—	848	950	1031	—

## Data Availability

All data from this case are accessible through the corresponding author and can be shared upon reasonable request. No publicly archived datasets were used or generated during the study.

## Nomenclature

CAD: coronary artery disease
CTEPH: chronic thromboembolic pulmonary hypertension
EKG: electrocardiogram
ED: emergency department
MRI: magnetic resonance imaging
NT-proBNP: N-terminal prohormone of brain natriuretic peptide
PFO: patent foramen ovale
PH: pulmonary hypertension
TEE: transesophageal echocardiogram
TTE: transthoracic echocardiogram
